# Factors associated with pregnancy-related anxiety among pregnant women attending antenatal care follow-up at Bedelle general hospital and Metu Karl comprehensive specialized hospital, Southwest Ethiopia

**DOI:** 10.3389/fpsyt.2022.938277

**Published:** 2022-09-23

**Authors:** Hunde Tarafa, Yadeta Alemayehu, Meskerem Nigussie

**Affiliations:** Department of Psychiatry, College of Health Sciences, Metu University, Metu, Ethiopia

**Keywords:** pregnancy-related anxiety, pregnant women, Bedelle, Metu, Ethiopia

## Abstract

Pregnancy-related anxiety (PRA) is an anxiety related to the pregnancy, involving labor and delivery, the well-being of the fetus/infant and the mother, the availability of quality of healthcare resources, and the capacity to parent. There is scarcity of study conducted on magnitude of Pregnancy-related anxiety and its associated factors among pregnant women in Ethiopia. The main objective of this research was to assess factors associated with Pregnancy-related anxiety among pregnant women attending ANC follow-up at Bedelle general and Metu Karl comprehensive specialized hospitals, Southwest Ethiopia. A hospital-based cross-sectional study design was used among pregnant women attending ANC follow-up. Data were collected from 406 sampled pregnant women who were selected through a systematic random sampling technique. Pregnancy-Related Anxiety Questionnaire-Revised (PRAQ-R2) was used to measure the outcome variable. The collected data were analyzed using Statistical Package for Social Sciences (SPSS) version 26. Logistic regression analyses were done to identify factors associated with Pregnancy-related anxiety and significance level set at *p* < 0.05. The overall prevalence of PRA in this study was 32.7%. Unwanted pregnancy AOR = 2.77, 95% CI [1.71, 4.54], high perceived stress AOR = 2.39, 95% CI [1.54, 3.62], young age AOR = 2.14, 95% CI [1.49, 2.83], depression AOR = 2.09, 95% CI [1.39, 2.89], low income AOR = 2.01, 95% CI [1.29, 3.14], and poor social support AOR = 1.79, 95% CI [1.14, 3.37] were significantly associated with Pregnancy-related anxiety. The findings of this study showed that the prevalence of Pregnancy-related anxiety was high in the study area and positively associated with young age, low income, poor social support, high perceived stress, depression, and unwanted pregnancy. This finding suggests that clinicians should integrate screening for Pregnancy-related anxiety into clinical standards, more efforts should be made in the future to reduce the anxiety among pregnant women who had an unwanted pregnancy, young women, and poor social support. Also, it is good to encourage the pregnant mother to enhance their social connectedness by creating a self-help group, and increasing early identification of mental health problems throughout their daily ANC follow-up.

## Introduction

Pregnancy is one of the most remarkable experiences in a woman's life. It is connected to a wide range of physical, enthusiastic, and social changes (1). Besides, pregnant women are preoccupied with fetal growth and future obligations, making them vulnerable to different psychological issues such as mood swings, exhaustion, mixed anxiety-depressive disorder, emotional disorders, and pregnancy-related anxiety (PRA) (2). PRA is a predominant issue for women both during and after pregnancy (3).

Pregnancy-related anxiety (PRA) is an anxiety related to the pregnancy, involving labor and delivery, the well-being of the fetus or infant, the well–being of the mother, the availability and quality of healthcare resources, and/or the capacity to parent (4–6). PRA is different from general anxiety that happens together with pregnancy (6) and has stronger associations to preterm birth (born at <37 weeks gestation) than more commonly researched general anxiety disorder or depressive disorders (4, 7–9). Preterm birth is thought to affect 85 percent of the world's population in Africa and Asia (10). Africa has the most prominent rate of preterm birth, with certain areas coming to 17.5%; generally 14.3% of births in Eastern Africa are preterm (10).

Untreated PRA may have a significant effect on the on the pregnant woman's and growing fetus' health (11). High levels of PRA have been connected to shorter gestation and duration of birth, miscarriage, and hypertension in pregnant women (4, 12, 13). Furthermore, high levels of PRA have been linked with preterm birth and low birth weight (4), as well as negative emotion (14, 15), attention-deficit hyperactivity disorder (ADHD), and delay in development (13, 16).

The prevalence of PRA has been reported to range from 6 to 29% in developed countries, with South Asia accounting for 32% (17–19). According to a systematic review study, the magnitude of PRA accounts from 1 percent to 26 percent in low- & middle-income nations (LMICs) (20). However, there appears to be a scarcity of published study on PRA in Ethiopia. Therefore, the purpose of this study was to determine the prevalence of PRA and its associated factors among pregnant women.

## Methods and materials

### Study setting and period

**Figure 1:**
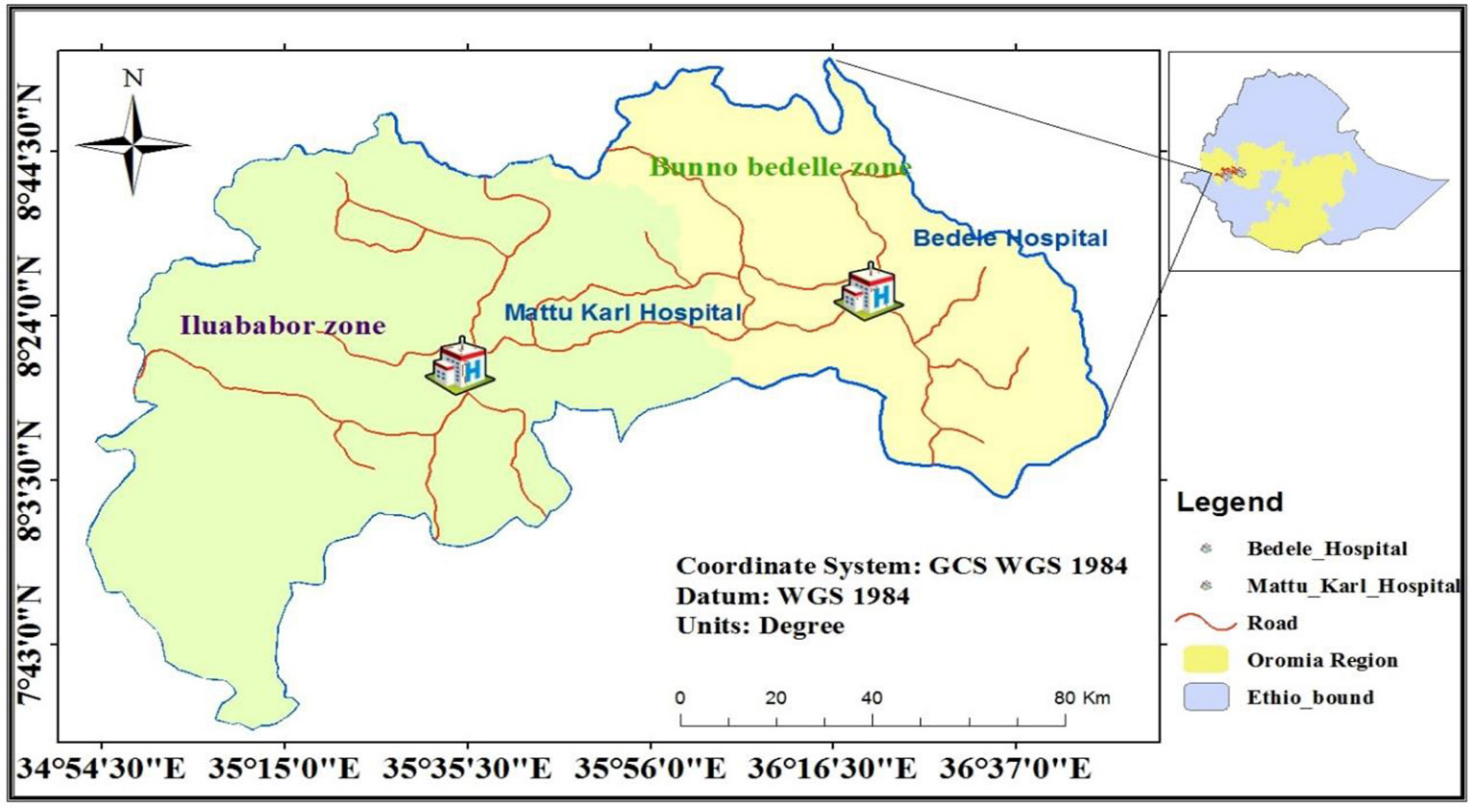
Study area map.

This study was conducted from September 14 to November 14 2021 at Bedelle general hospital and Metu Karl comprehensive specialized hospital. Bedelle general hospital is located in Bedelle town at a distance of 426 Km from Addis Ababa (see Figure 1). Bedelle general hospital was established in 2011GC. Bedelle general hospital has 8 gynecology bed rooms, three post-delivery rooms, and one Psychiatry outpatient department (OPD). It provides curative and rehabilitative services for about 1.5 million people living in Bunno Bedelle zone and people from different zones like east and west Wollega. Whereas Metu Karl comprehensive specialized hospital is found in Metu town, Ilu Abba Boor zone, Oromia region, Southwest Ethiopia (see Figure 1). Metu town is located 600 km far apart from the capital city of Ethiopia, Addis Ababa and 124 km away from Bedelle town. Its climatic condition is woynadega with full of forest and it is conducive environment for survival. Metu Karl comprehensive specialized hospital provides curative and rehabilitative services for about 2.1 million people living in Ilu Aba Bor zone and people from different regions like Gambella, Southern nation and nationalities peoples (SNNP) region and West Wollega. Also the hospital has four wards, 192 beds, two psychiatric OPD and giving different services as inpatient and outpatients and with different clinics.

### Study design and population

A hospital-based cross-sectional study was conducted among all sampled pregnant women attending ANC follow-up at Bedelle general hospital and Metu Karl comprehensive specialized hospital. Individuals who were critically ill during the data collection period and pregnant women aged <18 were excluded from the study since family consent is needed for their participation in the study.

### Sample size and sampling technique

The sample size was determined by the using single population proportion formula *n* = ${\left(Z_{\frac{\alpha }{2}}\right)}^{2}(\frac{p\left(1-p\right)}{d^{2}})$ (21) by assuming the proportion of pregnancy-related anxiety of 50% since there are no published studies in the study area, a confidence interval of 95, and 5% margin of error. Then, adding a non-response rate of 10%. Thus, the total sample size required was 423.

A systematic random sampling technique was used to select representative samples of pregnant women from both hospitals. Averagely 1,120 pregnant women attend ANC follow-up at both hospitals in 2 months. The sampling fraction was calculated by dividing the total number of pregnant women attending ANC follow-up in 2 months (1,120) by the final sample size (423); k = N/n = 2.6 ≈ 2. The first woman was selected by lottery method and then continued every 2 intervals. The data were collected from every other pregnant woman attending ANC follow-up at Bedelle general hospital and Metu Karl Specialized hospital.

### Variables of the study

The dependent variable in this study was pregnant-related anxiety. Independent variables included socio-demographic related variables (age, religion, marital status, ethnicity, level of education, educational status of the spouse, occupational status, occupational status of the spouse, family size, and income), psycho-social factors (family history of mental illness, social support, substance use history of the spouse, and marital satisfaction), health-related factors (current illness, depression, and perceived stress), pregnancy-related factors (duration of pregnancy, the woman's desire for pregnancy, and gravity), previous obstetrics factors (preterm history, stillbirth history, very low birth weight history, postnatal hemorrhage, abortion, and cesarean section), substance use-related factors (khat, alcohol, and tobacco).

### Data collection instruments

#### Pregnancy-related anxiety questionnaire-revised (PRAQ-R2)

The 10-item Pregnancy-Related Anxiety Questionnaire-Revised (PRAQ-R2) is a commonly used tool for assessing and identifying pregnancy-related anxiety in all pregnant women, regardless of parity. It has high psychometric values and predicts birth and childhood outcomes. In this study PRAQ-R2 adopted to measure the outcome variable. PRAQ-R2 contains 10 items. Each item asks about current feelings, and responses are measured on a 5-point likert scale: absolutely not relevant, hardly relevant, sometimes relevant, reasonably relevant, and very relevant, coded as “0”, “1”, “2”, “3”, and “4”, respectively. When all of the items are added together, the final score ranges from 0 to 40. “It consisted of three subscales: fear of giving birth, worries about a disabled child, and concern about one's appearance”. Scores of 13 or greater on PRAQ-R2 were considered as having pregnancy-related anxiety (22). In the current study the internal consistency (Cronbach's alpha) of PRAQ-R2 was 0.87.

#### Edinburgh postnatal depression scale (EPDS)

EPDS was used to screen for depression in this study. The EPDS is constituted of ten short statements. A mother selects one of four possible responses that best describe how she has felt over the last week. Responses are assigned a score of 0, 1, 2, or 3 based on the severity of the symptom. “Items 3, 5 to 10 are reverse scored (i.e., 3, 2, 1, and 0)”. The overall score is calculated by adding the scores for each of the 10 items. Mothers with a score of more than 13 were considered as depressed (23). In the current study the internal consistency (Cronbach's alpha) of EPDS was 0.84.

#### Perceived stress scale (PSS-10)

A perceived stress scale (PSS-10) with 10 items was used to collect data on perceived stress. The tool uses a 5-point Likert scale. Each item was rated using a scale of 0 = Never, 1 = Almost Never, 2 = Sometimes, 3 = Fairly Often, and 4 = Very Often. “The sum score ranged from 0 to 40 with higher scores indicating higher perceived stress”. “Scores ranging from 0 to 13 would be regarded as low stress, 14 to 26 would be considered moderate stress, and 27–40 would be considered high perceived stress” (24). In the current study the internal consistency (Cronbach's alpha) of PSS-10 was 0.93.

#### Oslo 3-items social support scale

Social support was measured by using Oslo social support questionnaire which has a score range from 3–14 that was interpreted as 3–8 is poor support, 9–11 is moderate support, and 12–14 is strong support (25). In the current study the internal consistency (Cronbach's alpha) of Oslo 3-items social support scale was 0.79.

#### Data collection procedures and data quality control

Interviewer-administered questionnaires were used to collect the data. The data were collected by five BSc. Nurses and supervised by two BSc. Psychiatry nurses. The questionnaire consisted of structured questions that can be subdivided into eight different categories: socio-demographic characteristics, PRAQ-R2, substance use, EPDS, PSS 10, psycho-social, health-related factors, and Oslo 3-items social support scale, previous obstetrics factors, and pregnancy-related factors.

The questionnaires were pretested on 5% (*n* = 21) of the total sample size at Chora primary hospital 1 week before data collection. Based on the pretest results, questions that were unclear or ambiguous were reviewed and fixed. The data collectors were supervised regularly, and the completed questionnaires were verified by the supervisor and principal investigator daily basis. To ensure consistency, the questionnaire was developed in English, and then translated into the local language Afan Oromo then back-translated into English by language experts. The data has been collected using the Afan Oromo version questionnaire.

#### Data processing and analysis

Data were coded and reviewed for completeness. Epi-data management version 4.6 was used to enter data, which was then exported to SPSS Version 26.0 for analysis. Tables and charts were used to present descriptive statistics such as frequency, percentage, mean, and standard deviation. The logistic regression analysis model was utilized; first, a bivariate analysis was performed to determine the association between each independent variable and pregnancy-related anxiety. Variables with *P* < 0.25 in bivariate analysis were incorporated into a multivariate logistic regression model to determine the association of each independent variable with pregnancy-related anxiety. *P*-value (*p* < 0.05) was used to determine statistical significance.

#### Ethical considerations

Ethical clearance was obtained from the ethical review committee of Metu University. A formal letter of permission was obtained from Bedelle general hospital and Metu Karl comprehensive specialized hospital. The study's objectives were explicitly conveyed to data collectors and study participants. The study participants were given the option to refuse or cease participation at any time, as well as the opportunity to ask any questions they had concerning the study. Participants' written consents were obtained before data were gathered. Throughout the study, confidentiality was maintained.

## Results

### Socio-demographic characteristics of participants

Four hundred six (406) study participants freely participated in the study, yielding a 96.0 percent response rate out of a total of 423 study participants. The majority 392 (96.53 %) of the study participants were married. The mean age of the participants was 28.4 (±6.13) years. Regarding educational level, 225 (55.4%) of the study participants were not attended formal education. The majority of respondents 360 (88.6%) were from the Oromo ethnic group, and 189(46.5%) were Orthodox Christian religion followers. Two hundred one (49.5 %) participants were housewives (Table 1).

**Table 1:** Socio-demographic characteristics of pregnant women attending ANC follow-up at Bedelle general hospital and Metu Karl comprehensive specialized hospital, 2021 (*N* = 406).

**Variables**	**Categories**	**Frequencies**	**Percent**
Age	18–23	41	10%
	24–29	119	29.3%
	30–35	139	34.2%
	>35	107	26.4%
Religion	Orthodox	189	47.7%
	Muslim	102	25.1%
	Protestant	112	27.5%
	Catholic	3	0.7%
Ethnicity	Oromo	360	88.6%
	Amhara	32	7.9%
	Tigre	10	2.5%
	Others	4	1.0%
Educational status	No formal education	225	55.4%
	Primary school	104	25.6%
	Secondary school	58	14.3%
	College and above	19	4.7%
Educational status of the spouse	No formal education	46	11.3%
	Primary school	151	37.2%
	Secondary school	107	26.4%
	College and above	88	21.7%
Marital status	Married	392	96.6%
	Unmarried	14	4.4%
Occupational status	Housewife	201	49.5%
	Trader	67	16.5 %
	Government employee	47	11.6%
	Daily laborer	42	10.4%
	Unemployed	34	8.4%
	Others	15	3.6%
Occupational status of spouse	Farmer	122	30.1%
	Trader	97	23.9%
	Government employee	78	19.2%
	Daily laborer	51	12.6%
	Unemployed	26	6.4%
	Others	18	4.4%
Family size	<5	252	62.1%
	>5	154	37.9%
Average family income	<3,000ETB	221	54.4%
	≥3,000ETB	185	45.6%

Other ethnicities: Gurage, Kaffa other occupations: student, farmer, religious.

### Psychosocial, substance use, and health-related characteristics of respondents

**Figure 2:**
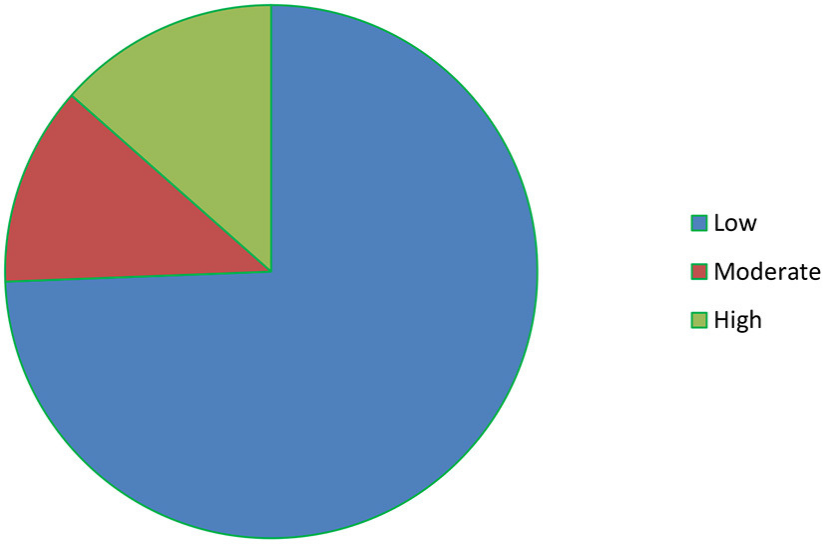
The level of perceived stress among respondents.

About half of the participants 204 (50.2%) had moderate social support; the majority of participants were reported a satisfying marital relationship 316 (77.8%) and about three fourth of the participants 302 (74.4%) had low perceived stress (Figure 2). Only 4.7% of the participants had a family history of mental illness. About one-third 118 (29.1%) of the participants had a spouse with an alcohol drinking habit and 42 (10.3%) had a spouse who smoke cigarettes (Table 2).

**Table 2:** Psychosocial, substance use, and health-related characteristics of pregnant women attending antenatal clinic follow-up at Bedelle general hospital and Metu Karl comprehensive specialized hospital, 2021 (*N* = 406).

**Variables**	**Categories**	**Frequencies**	**Percent**
Family history of mental illness	Yes	19	4.7
	No	387	95.3
Social support	Poor	82	20.2
	Moderate	204	50.2
	Strong	120	29.6
Current illness	Yes	54	13.2
	No	352	86.8
Marital satisfaction	Satisfying	316	77.8
	Not satisfying	90	22.2
Depression	Yes	121	29.8
	No	285	70.2
**Current substance use history of respondents**			
Khat	Yes	14	3.4
	No	392	96.6
Alcohol	Yes	31	7.6
	No	375	92.4
**Substance use history of the spouse**			
Khat	Yes	102	25.1
	No	304	74.9
Alcohol	Yes	118	29.1
	No	288	70.9
Tobacco	Yes	42	10.3
	No	364	89.7

### Previous obstetrics and pregnancy-related characteristics

Nearly three-quarters of participants 294 (72.4%) were multiparous; the majority of participants were in the second trimester of pregnancy 198 (48.7%) and the majority of participants 378 (93.1%) reported that the pregnancy was wanted. About 37 (9.1%) of participants had a history of abortion in the past (Table 3).

**Table 3:** Previous obstetrics and pregnancy-related characteristics of pregnant women attending antenatal clinic follow-up at Bedelle general hospital and Metu Karl comprehensive specialized hospital, 2021 (*N* = 406).

**Variables**	**Categories**	**Frequencies**	**Percent**
Duration of pregnancy	First trimester	118	29.1
	Second trimester	198	48.7
	Third trimester	90	22.2
The woman's desire for the pregnancy	Wanted pregnancy	378	93.1
	Unwanted pregnancy	28	6.9
Gravity	Primigravida	112	27.6
	Multigravida	294	72.4
Preterm history	Yes	12	2.9
	No	394	97.1
Stillbirth history	Yes	9	2.2
	No	397	97.8
Very low birth weight history	Yes	16	3.9
	No	390	96.1
Postnatal hemorrhage history	Yes	28	6.8
	No	378	93.2
Abortion	Yes	37	9.1
	No	369	89.9
Cesarean section	Yes	98	24.1
	No	314	75.9

### The prevalence of pregnancy-related anxiety among pregnant women

The overall prevalence of pregnancy-related anxiety in this study was 32.7 % with 95% CI (29.4, 36.3).

### Factors associated with PRA

#### Association of PRA with socio-demographic characteristics

The socio-demographic variables, including age, marital status, occupational status, and income have shown an association with pregnancy-related anxiety in bivariate logistic regression analysis. However, young age and low income were the only socio-demographic variables associated with pregnancy-related anxiety in the final model. See Table 4 for detail.

**Table 4:** Bivariate and Multivariate binary logistic regression analysis showing the association between pregnancy-related anxiety and socio-demographic variables among pregnant women attending ANC follow up Bedelle general hospital and Metu Karl comprehensive specialized hospital, 2021 (*N* = 406).

**Variables**	**Pregnancy-related anxiety**		**COR & 95% CI**	***P*-value**	**AOR & 95% CI**	***P*-value**
	**Yes**	**No**				
	***N* (%)**	***N* (%)**				
**Age**						
<24	24 (58.5)	17 (41.5)	3.31 (2.17–4.47)	0.001^*^	2.14 (1.49–2.83)	0.012^**^
≥24	109 (29.9)	256 (70.1)	1		1	
**Educational status of respondents**						
No formal education	79 (35.1)	146 (64.9)	1.65 (0.68–3.35)	0.311	
Primary school	35 (33.6)	69 (66.4)	1.54(.62–3.09)	0.416	
Secondary school and above	19 (24.7)	58 (75.3)	1	
**Educational status of the spouse**						
No formal education	17 (36.9)	29 (63.1)	1.12 (0.89–1.54)	0.499	
Primary school	49 (32.4)	102 (67.6)	0.92 (0.67–1.31)	0.646	
Secondary school and above	67 (34.4)	128 (65.6)	1	
**Marital status**						
Married	127 (32.4)	265 (67.6)	1	
Unmarried	6 (42.8)	8 (57.2)	1.56 (1.11–2.14)	0.030^*^	1.31 (0.97–1.84)	0.094
**Occupational status**						
Unemployed	16 (47)	18 (53)	1.94 (1.21–2.69)	0.001^*^	1.72 (0.95–2.98)	0.071
Employed	117 (31.5)	255 (68.5)	1		1	
**Occupational status of the spouse**						
Unemployed	11 (42.3)	15 (57.7)	1.47 (0.96–1.96)	0.294	
Employed	122 (33.3)	244 (66.7)	1	
**Family size**						
<5	85 (33.7)	167 (66.3)	1.1285 (0.79–1.68)	0.472	
>5	48 (31.2)	106 (68.8)	1	
**Average family income**						
<3,000ETB	86 (38.9)	135 (69.1)	1.87 (1.39–2.51)	<0.001^*^	2.01 (1.29–3.14)	<0.001^**^
≥3,000ETB	47 (25.4)	138 (74.6)	1		1	

* Shows significance in bivariate analysis;

** significant in multivariate regression.

The odds of PRA were 2.14-times (AOR = 2.14, 95% CI = 1.49, 2.83) higher in pregnant women aged <24 compared to those aged 24 and above. Similarly, pregnant women who reported low family income were about 2-times more likely to have pregnant-related anxiety than pregnant women who have good family income AOR = 2.01, 95% CI (1.29, 3.14).

#### Association of pregnancy-related anxiety with psychosocial factors, substance use history, and health-related variables

From psychosocial factors, substance use history and health-related variables, family history of mental illness, social support, perceived stress, depression, current illness, khat use of the respondent, alcohol use of the respondent, alcohol use history of the spouse, and tobacco use history of the spouse were associated with pregnancy-related anxiety in bivariate logistic regression analysis. However, poor social support, high perceived stress, and depression were psychosocial and health related variables associated with pregnancy-related anxiety in the final model. See Table 5 for detail.

**Table 5:** Bivariate and Multivariate binary logistic regression analysis showing the association between PRA and psychosocial, substance use, and health-related variables among pregnant women attending ANC follow-up Bedelle general hospital and Metu Karl comprehensive specialized hospital, 2021 (*N* = 406).

**Variables**	**Pregnancy-related anxiety**		**COR & 95% CI**	***P*-value**	**AOR & 95% CI**	***P*-value**
	**Yes**	**No**				
	***N* (%)**	***N* (%)**				
**Family history of mental illness**						
Yes	9 (47.3)	10 (52.7)	1.91 (1.13–2.93)	0.031^*^	1.64 (0.89–2.63)	0.089
No	124 (32)	263 (68)	1		1	
**Social support**						
Poor	37 (45.1)	45 (54.9)	2.36 (1.59–4.17)	<0.001^*^	1.79 (1.14–3.37)	<0.001^**^
Moderate	65 (31.9)	139 (68.1)	1.34 (0.76–2.33)	0.314	
Strong	31 (25.8)	89 (75.2)	1		1	
**Current illness**						
Yes	24 (44.4)	30 (55.6)	1.78 (1.22–3.01)	0.041^*^	1.29 (0.83–2.12)	0.131
No	109 (31)	243 (69)	1	
**Marital satisfaction**						
Yes	96 (28.5)	226 (71.5)	1	
No	31 (34.4)	59 (65.6)	1.24 (0.71–2.10)	0.320	
**Perceived stress**						
Low	88 (29.1)	214 (60.9)	1	
Moderate	19 (38.8)	30 (61.2)	1.54 (1.13–2.04)	0.089^*^	
High	26 (47.3)	29 (52.7)	2.18 (1.37–3.22)	<0.001^*^	2.39 (1.54–3.62)	<0.001^**^
**Depression**						
Yes	56 (46.3)	65 (43.7)	2.33 (1.55–3.47)	<0.001^*^	2.09 (1.39–2.89)	<0.001^**^
No	77 (27)	208 (73)	1	
**Substance use history of the respondents**						
**Khat**						
Yes	6 (42.8)	8 (57.2)	1.56 (1.01–2.73)	0.135^*^	1.19 (0.63–1.94)	0.711
No	127 (32.4)	265 (67.6)	1			
**Alcohol**						
Yes	14 (45.2)	17 (54.8)	1.77 (0.99–3.10)	0.051^*^	1.48 (0.71–2.67)	0.338
No	119 (31.7)	256 (68.3)	1			
**Substance use history of the spouse**						
**Khat**						
Yes	37 (36.3)	65 (63.7)	1.23 (0.76–1.98)	0.414		
No	96 (31.6)	208 (68.4)	1			
**Alcohol**						
Yes	51 (44.1)	67 (56.9)	1.91 (0.99–3.10)	0.064^*^	1.63 (0.91–3.14)	0.103
No	82 (28.5)	206 (71.5)	1	
**Tobacco**						
Yes	19 (45.2)	23 (54.8)	1.81 (1.14–3.06)	0.039^*^	1.35 (0.83–2.17)	0.087
No	114 (31.3)	250 (68.7)	1	

* Shows significance at bivariate analysis;

** shows significance at multivariate analysis.

In this study, the odds of having PRA among respondents who have poor social support were 1.79-times (AOR = 1.79, 95% CI = 1.14, 3.37) higher compared to those who have strong social support. Likewise high perceived stress increases the likelihood of pregnancy-related anxiety by 2.4-times as compared to low perceived stress, AOR = 2.39, 95% (1.54, 3.62). The odds of having pregnancy-related anxiety among respondents who have depression symptoms were 2.01-times higher than those without depression symptoms (AOR = 1.84, 95% CI = 1.01–3.35).

#### Association of pregnancy-related anxiety with previous obstetrics and pregnancy-related factors

From previous obstetrics and pregnancy related variables, unwanted pregnancy, preterm history, gravity, history of abortion, and history of cesarean section delivery were associated with pregnancy-related anxiety in bivariate logistic regression analysis. However, unwanted pregnancy was the only pregnancy-related variable associated with pregnancy-related anxiety in the final model.

In this study, the odds of having pregnancy-related anxiety among respondents who had reported unwanted pregnancy was 2.77-times (AOR = 2.77, 95% CI = 1.71, 4.54) higher as compared to those who reported the pregnancy is wanted. See Table 6 for detail.

**Table 6:** Bivariate and Multivariate binary logistic regression analysis showing the association between PRA and previous obstetrics and pregnancy related variables among pregnant women attending ANC follow up Bedelle general hospital and Metu Karl comprehensive specialized hospital, 2021 (*N* = 406).

**Variables**	**Pregnancy-related anxiety**		**COR & 95% CI**	***P*-value**	**AOR & 95% CI**	***P*-value**
	**Yes**	**No**				
	***N* (%)**	***N* (%)**				
**Duration of pregnancy**						
First trimester	35 (26.7)	83 (73.3)	1	
Second trimester	69 (34.8)	129 (65.2)	1.26 (0.71–2.11)	0.359	
Third trimester	29 (32.2)	61 (37.8)	1.13 (0.54–2.23)	0.624	
**The woman's desire for pregnancy**						
Wanted pregnancy	116 (30.7)	262 (69.3)	1		1	
Unwanted pregnancy	17 (60.7)	11 (29.3)	3.49 (2.15–4.93)	<0.001^*^	2.77 (1.71–4.54)	<0.001^**^
**Gravity**						
Primigravida	55 (49.1)	57 (50.9)	2.67 (1.51–3.47)	0.021^*^	1.33 (0.78–1.94)	0.413
Multigravida	78 (26.5)	216 (73.5)	1	
**Preterm history**						
Yes	5 (41.7)	7 (48.3)	1.48 (0.88–2.72)	0.192^*^	1.10 (.72-1.65)	.620
No	128 (32.5)	266 (67.5)	1	1
**Postnatal hemorrhage history**						
Yes	11 (39.3)	17 (60.7)	1.35 (0.64–2.01)	0.621		
No	122 (32.3)	256 (67.7)	1			
**History of abortion**						
Yes	16 (43.2)	21 (56.8)	1.64 (0.96–1.84)	0.083^*^	1.21 (0.82–1.84)	0.149
No	117 (31.7)	252 (68.3)	1		1	
**History of cesarean section**						
Yes	39 (39.8)	59 (60.2)	1.55 (1.01–3.33)	0.045^*^	1.39 (0.81–2.73)	0.151
No	94 (29.9)	220 (70.1)	1		1	

* Shows significance at bivariate analysis;

** significant at multivariate analysis.

## Discussion

The purpose of this study was to determine the prevalence of PRA and its associated factors among pregnant mothers receiving antenatal care at Bedelle general hospital and Metu Karl comprehensive specialized hospital. The magnitude of PRA among pregnant mothers in this research was 32.7% (95% CI 29.4, 36.3). The finding of our study was similar with the finding of the research that undertaken in Dilla university referral hospital where 32.2% of the study participants were screened positive for anxiety (26). However, The reported prevalence rates from other studies using different scales appear to be much lower than the finding of this study: 10.04 percent in Arba minch area district study by utilizing the General Anxiety Disorder 7-items to screen PRA (27), 25 percent in Tanzania study in which they used PRAQ to screen PRA (28), 23 percent in South Africa in which they utilized the Mini-International Neuropsychiatric Interview diagnostic interview to screen PRA, 23.6 percent in Saudi Arabia study using the State Anxiety scale (29), 26.6 percent in Qatar study using PRAQ-R2 (22), and 26.8 percent in Brazil study using the Hospital Anxiety Subscale (30). In contrast, the result of this study is lower than the result of the study that conducted in southern India (31) where 55.7% pregnant mothers have screened positive for PRA.

This variation in prevalence of PRA could be related to discrepancies in the psychometric qualities of the measuring tool utilized. In addition it might be related to demographic and sociocultural differences. Societal norms and beliefs can also influence views of what is “stressful” or “hazardous,” accounting for the diversity in prevalence across different study contexts. Moreover, other possible reasons for the difference in prevalence can be related to variation in sample size and sampling techniques.

Our study showed that being pregnant at young age was found to be a risk factor for PRA. The finding of this research is similar with the finding of a study conducted in in India (32), Hungary (33), and Sweden (19). This association might be attributed to an increased feeling about the fear of birth and approaching birth may leads to the development of pregnancy-related anxiety. The possible reason could be that at an early age, the pregnant mother might anxious the possibility of fetus loss and the viability of a developing fetus.

In this study, low income was significantly associated with pregnant-related anxiety. This is similar with the result of studies undertaken in United States (34) and Hungary (33). These findings show that low-income pregnant women may be obsessed with issues associated with parenting, such as higher financial expenses, availability to prenatal service, and maternity leave. For instance, the anticipated financial costs of maintaining a baby may be an extra pressure during the perinatal time, influencing the occurrences of anxiety. Additionally, pregnant mother with low financial income may be worried about their capability to provide for their child; as a result of this, they develop higher level of anxiety symptoms. Low income pregnant mother may be worried about their access to ANC service, which may be hindered by their family income. Pregnant mother with lower socioeconomic status receive limited prenatal service at higher rates than their middle and high socioeconomic status counterparts (35), and lower socioeconomic status has been linked with high risk of hypertension, diabetes, and other complications related to pregnancy (35).

Our study finding indicated that those pregnant mothers who have poor social support had higher chance of PRA compared to pregnant mothers who have strong social support. This is similar to the results of the researches that undertaken in Dilla university referral hospital (26), Hungary (33), and Australia (36). Pregnant mothers who have poor social support might not happy with their family and has no good interaction with their social environment, and due to this, they may develop social withdrawal, becoming less psychological and coping ability and eventually leads to more anxiety (37).

This research has indicated that depression is highly linked with pregnancy-related anxiety. The existing research supports the established association between depression and pregnancy-related anxiety (6,40). Pregnant mother have high chance of suffering anxiety and depression than non-pregnant women (38, 39). However, there appears to be a lack of knowledge as to why this comorbidity emerges and whether depression or anxiety develops first in this inquiry, stress, appraisal, and coping theory consideration (40), along with the intricacies of this sample's sociocultural circumstances, gives insight. According to Lazarus and Folkman (40), stress happened when an event is seen as a potential danger to one's well-being, and the assets to play down the occasion are respected as inaccessible. Furthermore, conditions such as uniqueness and closeness to the situation can influence perceptions of the severity of the danger happened (40). According to this concept, an inadequate basic health related knowledge, combined with constrained get to healthcare facilities and a destitute financial status, makes an environment in which pregnancy is frequently linked with stress or anxiety (41).

In the current study high level of perceived stress is strongly associated with pregnancy-related anxiety. This findings is similar with the finding of a study that conducted in Tanzania (28). Perceived stress is a person thought of the level of suffering posed by stressors, and their capability to cope with those stressors. Pregnant mothers usually develop a high level of perceived stress, due to the stress of approaching the delivery time; this may in turn leads to anxiety (41).

This research showed that unwanted pregnancy was a significant predictor of PRA. This finding is consistent with a findings of researches conducted in Nigeria (42), Spain (43), and China (44). This association is possible explained lack of financial preparation from women to have a new baby and lack of psychological preparedness for motherhood (44–46). Moreover, an unwanted pregnancy was linked to increased levels of anxiety during the delivery process.

This research has number of limitations. First, the cross-sectional design of our study limits the ability to draw conclusions about directions of the relationships. Second, since the study is limited to Bedelle general hospital and Metu Karl comprehensive specialized hospital, the findings of research may not inferred to health facilities. Some variables were assessed with only self-report such as family history of mental illness, pregnancy wanted or unwanted, and medical illness which may have influenced by social desirability bias. We attempt to minimize these limitations by giving training for the data collectors to explain the respondents the objective of the study and interviewing them in to the way their privacy is maintained and informing them as the information collected from them was anonymous and this may in turn increase the response rate.

## Conclusion

The magnitude of pregnancy-related anxiety among pregnant mothers in the study setting was high. One in three pregnant women was found to have PRA in the study setting. Young age, low income, poor social support, high perceived stress, depression, and unwanted pregnancy were significantly associated with PRA. The clinicians working in ANC need to include mental health screening in their routine services and forward the right intervention to initiate and enhance access to mental health treatment, especially for vulnerable groups. Early treatment and integrating mental health services for co-morbid mental illness and ANC is essential, since PRA is notably common among pregnant mothers. It is better if interventions focus among the pregnant mothers who had a history of depression and mothers with unwanted pregnancy are implemented. In addition, it is better to encourage the pregnant women to increase their social network by establishing a self-help group, and strengthening early detection of mental health problems throughout their routine ANC follow-up. Based on the study findings, we recommend that the concerned public organizations like women and youth affair and other governmental organization (NGO) work on educating women on preventing unwanted pregnancy as well as policy makers consider stress management technique to ANC to curb the burden on pregnant women.

## Data availability statement

The raw data supporting the conclusions of this article will be made available by the authors, without undue reservation.

## Author contributions

HT conceived the study design, collected, analyzed, interpreted data, and drafted the manuscript for important intellectual content. YA and MN conceived the study design, interpreted data, and review the manuscript for important intellectual content. All authors contributed to the article and approved the submitted version.

## Conflict of interest

The authors declare that the research was conducted in the absence of any commercial or financial relationships that could be construed as a potential conflict of interest.

## Publisher's note

All claims expressed in this article are solely those of the authors and do not necessarily represent those of their affiliated organizations, or those of the publisher, the editors and the reviewers. Any product that may be evaluated in this article, or claim that may be made by its manufacturer, is not guaranteed or endorsed by the publisher.

## Abbreviations

ANC: Antenatal Care
AOR: Adjusted odds ratio
COR: Crude odds ratio
EPDS: Edinburgh Postnatal Depression Scale
LMICs: Low-income and Middle-Income Countries
PRA: Pregnancy-related anxiety
PRAQ-R2: Pregnancy-Related Anxiety Questionnaire-Revised
PSS-10: Perceived Stress Scale
SPSS: Statistical Package for Social Sciences.
